# Aging increases lightness of grass-fed beef

**DOI:** 10.1093/tas/txae140

**Published:** 2024-09-25

**Authors:** Jordan C Wicks, Alexis L Wivell, Mariane Beline, Morgan D Zumbaugh, Jocelyn S Bodmer, Con-Ning Yen, Thomas B Wilson, Scott P Greiner, Sally E Johnson, Tim H Shi, Saulo L Silva, David E Gerrard

**Affiliations:** School of Animal Sciences, Virginia Polytechnic Institute and State University, Blacksburg, VA 24061, USA; School of Animal Sciences, Virginia Polytechnic Institute and State University, Blacksburg, VA 24061, USA; School of Animal Sciences, Virginia Polytechnic Institute and State University, Blacksburg, VA 24061, USA; School of Animal Sciences, Virginia Polytechnic Institute and State University, Blacksburg, VA 24061, USA; School of Animal Sciences, Virginia Polytechnic Institute and State University, Blacksburg, VA 24061, USA; School of Animal Sciences, Virginia Polytechnic Institute and State University, Blacksburg, VA 24061, USA; School of Animal Sciences, Virginia Polytechnic Institute and State University, Blacksburg, VA 24061, USA; School of Animal Sciences, Virginia Polytechnic Institute and State University, Blacksburg, VA 24061, USA; School of Animal Sciences, Virginia Polytechnic Institute and State University, Blacksburg, VA 24061, USA; Department of Animal Science and Food Engineering, College of Animal Science and Food Engineering, University of SaoPaulo, Pirassununga, SP, 13635-900, Brazil; School of Animal Sciences, Virginia Polytechnic Institute and State University, Blacksburg, VA 24061, USA

**Keywords:** beef quality, color, dark beef, dry aging, grass-fed, muscle type

## Abstract

Grass-fed beef is becoming increasingly popular and is expected to be a $14 billion industry by 2024. Even so, grass-fed beef is typically darker in appearance than that of conventional grain-fed beef. Aging has been shown to improve lean color (L*, a*) of dark-cutting beef however little work has focused on aging as it relates to improving the lean color of grass-fed beef. Therefore, the aim of this study was to evaluate the effect of dry aging on grass-fed beef compared to varying lengths of grain-feeding. Thirty commercial Angus crossbred steers were randomly assigned to either pasture finishing (CON), short (SF), or long fed (LF) programs. The SF and LF treatments ranged from 90 to 114 d (average: 98 d) and 118 to 134 d (average: 125 d) on ad libitum high concentrate feeding, respectively. Cattle were randomly identified from each treatment group and harvested over a consecutive 3-wk span. Carcass evaluation and longissimus lumborum samples were collected 24 h postmortem. Carcasses were aged for 21 d, and steaks were collected on 1-, 3-, 7-, 10-, 14-, and 21-d postharvest, and objective color was evaluated following 1 h bloom. Our data show color (L*, a*, b*) was improved with aging regardless of treatment. However, grass-fed (CON) showed the greatest improvement in both lightness (L*) and redness (a*) ultimately making grass-fed comparable to that of grain-fed beef by day 21. These data argue that dry-aging grass-fed beef improves color development similar to that of grain-fed beef.

## Teaser

Aging improves the lean color of grass-fed beef.

## Introduction

Currently, grass-fed beef represents approximately 4% of the US beef industry and is estimated to contribute approximately $4 billion to the overall US beef industry (Bauman, 2021). Though only a minor contributor to the overall US beef industry, demand for grass-fed beef continues to grow. In fact, the global grass-fed beef movement is projected to become a $14 billion industry by 2024, with the US contributing to nearly 50% of the market (Chase, 2020). This projection illustrates a shift in consumer trends and the future of the US beef industry. While an attractive alternative to conventionally raised beef for socially conscious consumers, there are negative attributes associated with beef produced in these systems. In particular, grass-fed beef is typically darker in appearance and less tender compared to its grain-fed counterpart (Crouse et al., 1984; reviewed by Brewer et al., 2003; Chail et al., 2016; Apaoblaza et al., 2020; Gómez et al., 2022). This attribute significantly detracts from the perceived freshness by consumers and limits marketing options (Martin and Rogers, 2004).

Animals subjected to low-energy, high-forage-based diets tend to have a more oxidative muscle metabolism and as such, this characteristic partially contributes to dark lean arising from grass-fed beef (Apaoblaza et al., 2020; Antonelo et al., 2022). However, when weight and backfat are held constant, variations in quality dissipate between grass and grain-finished beef (Davis et al., 1981; Wicks et al., 2019). While interesting, the practical application of exploiting this mitigation strategy is nearly impossible because the feeding regime is confounded by growth rate, and therefore changes in rates of gain and animal age vary with the feeding strategy. Thus, identification of a postharvest process that improves the lean color of grass-fed beef is highly coveted.

Aging meat is well-established in increasing tenderness through the natural process of proteolysis (reviewed by Veiseth and Koohmaraie, 2005), and while not a direct approach to mitigate dark lean, aging also improves lean color (reviewed by Suman et al., 2014). However, to our knowledge, no studies have investigated the use of dry aging to improve color in grass-fed beef and only one has investigated wet-aging approaches to improve color development in grass-fed beef (Holman et al., 2022). While promising, this process is aimed at larger production systems that cut, vacuum package, and ship products rapidly. Though useful, much of the grass-fed movement in the US involves local direct marketing outlets, which utilize the services of small and very small processors. Therefore, given the need for rapid turnover and increased warehouse storage required by wet-aging protocols, these practices are not typically feasible for small processors. Yet, the practice of dry-aging beef is becoming more widely accepted by both processors and consumers and may be a practical solution for the niche grass-fed market. Therefore, the aim of this study was to understand the effect dry aging has on grass-fed beef color development compared to grain-fed beef.

## Materials and Methods

All animal experimental procedures were approved by the Virginia Tech Institutional Animal Care and Use Committee under protocol number 18-180. Experimental animals were managed according to the guidelines outlined in the Guide for the Care and Use of Agricultural Animals in Research and Teaching (FASS, 2020).

### Animals and Treatments

**Figure 1.**
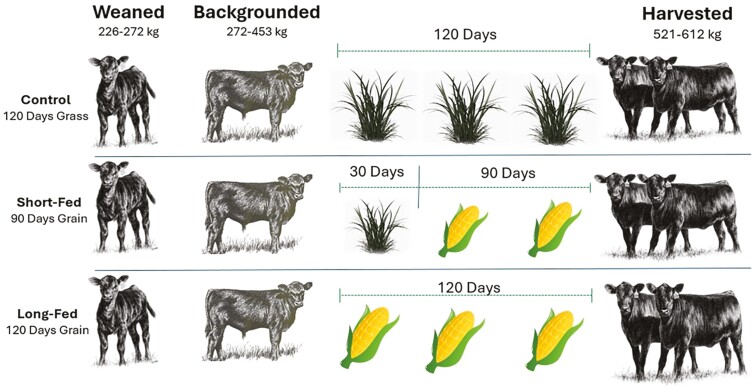
Schematic diagram of feeding regime for cattle subjected to control (CON), short-fed (SF), or long-fed (LF).

Thirty Angus crossbred steers of similar body weight (BW) and age (BW = 262 ± 22 kg, age = 7.9 mo ± 2 mo) were sourced after weaning from the Virginia Tech Beef Center in Blacksburg, VA. All steers were grazed on tall fescue pastures during a 214 d backgrounding period. The backgrounding period lasted 214 d from fall 2020 to spring 2021 with cattle-fed alfalfa silage in concrete bunks during the winter. On reaching an average BW of 442 ± 37 kg, cattle were randomly assigned to 1 of 3 finishing programs (Fig. 1) 1) pasture finishing on tall fescue pastures without supplementation for 127 ± 6 d (CON, *n* = 10), 2) a short grain-finishing period (SF, *n* = 10) that grazed cattle on pasture for 30 d and then transitioned them to a grain-based diet, or 3) a long grain-finishing period (LF, *n* = 10) that fed cattle on a grain-based diet for the duration of the finishing period. Steers of treatment groups (*n* = 10) were reared in separate, but adjacent paddocks (Gootwine et al., 2020; Wicks et al., 2024). Cattle were harvested at the Virginia Tech Meat Center beginning on day 119 of the finishing period. Steers were harvested over a 26-d period in 9 groups. Specifically, one animal from each treatment per harvest group, with the exception of one harvest group that had having 2 animals per treatment combination Treatments were randomly allotted to harvest groups with an equal number of cattle from each treatment harvested each week. Length of the finishing period was not different (*P* = 0.702) between treatments with CON, SF, and LF being finished over 127 ± 6, 128 ± 8, and 125 ± 6 d periods, respectively.

Steers allotted to SF and LF were fed the same grain-based finishing diet that contained 38.7% cracked corn, 38.5% pelleted corn gluten feed, 20.2% corn silage, 1.0% custom mineral mix, and 1.6% ground limestone (Table 1). The grain-finishing diet was limit-fed to 16.7 kg/d and formulated using the NASEM (2016) model with a target metabolizable energy allowable gain of 1.59 kg/d. Two transition diets were used over a 10-d period to transition LF and SF cattle to the finishing diet beginning on days 0 and 30 of the finishing period, respectively.

**Table 1.** Grain-finishing diet fed to short- and long-fed market steers

Item	Finishing diet^1^
Ingredient, % DM	
Cracked corn	38.7
Pelleted corn gluten feed	38.5
Corn silage	20.2
Custom mineral^2^	1.0
Ground limestone	1.6
Analyzed nutrient content	
DM, % as fed	67.3
CP, % DM	13.0
NDF, % DM	25.1
ADF, % DM	11.0
NE_m_, Mcal/kg	1.84
NE_g_, Mcal/kg	1.21
DMI, kg/d	16.7

^1^Finishing diet was fed to long-fed (LF) cattle from days 0 to 125 ± 6 of finishing period. Short-fed (SF) cattle were moved from pasture on day 30 of finishing period and placed on a finishing diet for 98 ± 8 d (128 ± 8 d total finishing period) and to SF cattle from day 30 of finishing period to slaughter.

^2^Custom mineral (Southern States Cooperative, Richmond, VA) contained: 18.5% salt, 9.35% Ca (CaCO_3_ and CaHPO_4_), 2.0% P (CaHPO_4_), 12.0% Mg (MgO and MgSO_4_), 1.0% K (KCl and K_2_SO4), 0.5% S (K_2_SO4, MgSO_4_, FeH_2_O_5_S, and CoSO_4_), 3,000 mg/kg Zn (ZnO), 1,200 mg/kg Mn (MnO₂), 1,200 mg/kg Cu (C_4_H_16_Cl_2_CuN_4_), 60 mg/kg Se (Na_2_SeO_3_), 60 mg/kg I [Ca(IO_3_)_2_], 50 mg/kg Co (CoCO_3_ and CoSO_4_), 220,462 IU/kg vitamin A, 55,115 IU/kg vitamin D, and 551 IU/kg vitamin E.

During the first 30 d of the finishing period, CON and SF steers were rotated through the same endophyte-infected tall fescue 12.3 ha pasture system at a stocking density of 0.615 ha/steer. After SF cattle were transitioned to the grain-finishing diet, CON remained in the same pasture system at a stocking density of 1.23 ha/steer. Cattle were rotated to new paddocks when standing forage dry matter (DM) was visually estimated to 454 kg/ha. Grazing cattle had ad libitum access to mineral and automatic waterers. Grain-fed treatments were housed in two 0.40 ha drylot pens (10 steers per pen). Drylot pens were open, unpaved lots with a row of trees to provide a windbreak. Cattle were fed once daily in concrete, fence line bunks. Pen and feed bunk space exceeded FASS (2020) guidelines. Cattle had ad libitum access to automatic waterers.

### Animal Growth Performance Measures

Steer BW was recorded at the beginning of the backgrounding period as well as days 0, 30, 60, and 86 of the finishing period. Final BW was recorded at the Virginia Tech Beef Center on the day steers were transported to the Virginia Tech Meat Center for harvest the following day. For grain-fed cattle, an empty BW was recorded before feeding. Average daily gain (ADG) was calculated for the 214-backgrounding period, days 0 to 30 of finishing, days 30 to 60 of the finishing period, day 30 to harvest, and for the entire 127 ± 7 d finishing period. The BW recorded on day 0 of the finishing period was used as the final backgrounding BW when calculating ADG for the backgrounding period.

### Harvest and Tissue Sample Collection

All cattle were harvested under Virginia Department of Agriculture Consumer Services meat and poultry inspection. Standard industry harvesting procedures were used, and all carcasses entered a conventional chilling cooler (2 ± 1 °C) at approximately 50 min postmortem.

Tissue from the longissimus lumborum (LL) was excised immediately following exsanguination (0 min) as well as at 3, 6, 12, and 24 h postmortem. Samples were diced, snap-frozen in liquid nitrogen, and stored at −80 °C until analyses. In addition, whole bone-in loins were fabricated from all carcasses following a 24-h chilling period and aged for 21 d and described below.

### Carcass Evaluation

Following a 24-h chilling period (2 ± 1 °C) carcasses were ribbed between the 12th and 13th rib for carcass evaluation as described by the American Meat Science Association (AMSA, 2001) Ribeye area (REA), 12th rib back fat thickness, estimated percent kidney, pelvic and heart fat (KPH), and hot carcass weight (HCW) were measured and used to calculate carcass yield grade. Carcass maturity and marbling scores were used to determine carcass quality grade.

### Color Analysis

Color development was measured over time using steaks from aged loins. Briefly, bone-in loins were removed from carcasses following a 24-h chilling period, placed in a chilling cooler (2 ± 1 °C), and dry aged for 21 d. One 2.54-cm thick steak was randomly collected from each loin on designated aging timepoints (1, 3, 7, 10, 14, and 21 d). Steaks were allowed 1 h to bloom and objective color was evaluated at a bloom temperature of 3 ± 1 °C. Using a Minolta CR-400 colorimeter (Ramsey, NJ, USA), Illuminant D, 0° observer angle, triplicate color measurements were taken, and averaged color values were expressed as Commission Internationale de l’Éclairage (CIE) L* (lightness), a* (redness), and b* (yellowness).

### pH Analysis

Muscle pH was determined using the iodoacetic method, as described by Bendall (1973) with some modifications. Briefly, powdered muscle and buffer (1:8 w/v) were homogenized using a Qiagen TissueLyser II (2 min at 25 Hz). Homogenization buffer contained 5 mM Na-iodoacetic acid and 150 mM KOH. Once homogenized, samples were heated to 25 °C for 5 min, centrifuged for 5 min at 13,000 × *g*, and placed back on the heating block at 25 °C for 1 min. pH was measured using a calibrated Orion Ross Ultra pH electrode (Thermo Scientific, Pittsburgh, PA).

### Protein Extraction and Determination

Finely powdered tissue was homogenized (TissueLyzer II; Qiagen, USA; 2 min 25 Hz) with a buffer containing 8 M urea, 2 M thiourea, 3% SDS (w/v), 75 mM DTT, 0.05 M Tris–HCl (pH 6.8) heated at 95 °C (Warren et al., 2003). Samples were then diluted at 1:20 and used for total protein quantification using Reducing Agent and Detergent Compatible Protein Assay (Bio-Rad Laboratories, Hercules, CA, USA), according to manufacturer’s specifications. Finally, samples were diluted to a final concentration of 3 mg/mL in extraction buffer (Warren et al., 2003) containing 0.05% bromophenol blue. An additional tube of 100 mg tissue was homogenized with an extraction buffer containing 50 mM Tris HCl, 150 mM NaCl, 1% NP, 0.25% sodium deoxycholate. Samples were centrifuged at 10,000 × *g* for 10 min and supernatants were collected. Homogenates were diluted 1:20 and protein concentration was determined using BCA protocol. Samples were then diluted in extraction buffer and Lamelli buffer to a final concentration of 3 mg/mL. All samples were allocated and stored at −80 °C until further analysis.

### SDS–Page and Immunoblotting

Samples were heated to 60 °C for 10 min prior to being subjected to SDS–PAGE. A stacking gel of 5% was used for all proteins, however separating gels varied on protein of interest. A 15% polyacrylamide separating gel was used to detect succinate dehydrogenase (SDH; Abcam ab14715 at 1:1000 dilution) and lactate dehydrogenase (LDH; Novus NBPI48336 at 1:30,000 dilution). For detection of citrate synthase (CS; Santa Cruz Biotechnology, Inc, SC-390693 at 1:1000 dilution), phosphofructokinase (PFK, Santa Cruz Biotechnology, Inc, SC-166722 at 1:1000 dilution), adenosine monophosphate deaminase 1 (AMPD1; Abcam Ab72541 at 1:1000 dilution) a 10% gel was used, while an 18% gel was used for myoglobin (Santa Cruz Biotechnology, Inc, SC-25607 at 1:1000 dilution). Gels were allowed to run at room temperature at 60 V for 20 min, and then 120 V for 120 min (Bio-Rad Laboratories) for SDH, LDH, and CS. Gels for PFK and AMPD1 were also run at room temperature. However, AMPD1 and PFK gels were run at 50 V for 25 min, then 100 V for 150 min, while myoglobin gels were electrophoresed at 60 V for 20 min and 200 V for 50 min (Bio-Rad Laboratories). Gels were then transferred to nitrocellulose membranes at 70 V for 50 min at 4 °C using a Bio-Rad (Bio-Rad Laboratories) and a transfer buffer containing 50 mM Tris, 0.38 M glycine, 0.01% (w/v) SDS, and 10% (v/v) methanol. Membranes were blocked overnight at room temperature with either Prometheus OneBlock Blocking buffer (Genesee Scientific Corporation, El Cajon, CA) or 5% nonfat dry milk in Tris-buffered saline solution with 0.1% tween-20 (1X TBS-T) added. Primary antibodies specific for SDH, LDH, CS, PFK, AMPD1, and myoglobin were diluted in respective blocking buffers and incubated overnight at room temperature on membranes. Membranes were then washed with TBS-T 3 times for 5 min each before incubated for 1 h at room temperature with a secondary antibody diluted in TBS-T. The goat anti-mouse fluorescent antibody (LI-COR Biosciences, Lincoln, NE) was used for SDH, while the goat anti-rabbit fluorescent antibody (LI-COR Biosciences) was used for LDH. Membranes were washed an additional 3 times for 5 min each. All membranes were reversibly stained with Revert 700 Protein Stain (LI-COR Biosciences., Lincoln, NE) for visualization of bands, and all targeted proteins were normalized to total protein. All blots were imaged using a LI-COR Biosciences Odyssey Infrared scanner (Li-Cor, Inc.), and band intensity was measured using Image Studio Lite (Li-Cor, Inc.) with protein abundance reported as arbitrary units.

### Gene Expression

Direct-zol RNA Mini Prep Kit (Zymo Research, Irvine, CA) was used to extract total RNA from 0 min LL muscle. About 20 ng/µL of total RNA was reverse transcribed using the High Capacity cDNA Reverse Transcriptase Kit (Applied Biosystems, Waltham, MA). Two microliters of cDNA were amplified using gene-specific primers (Table 2) and SYBR chemistry in a 7500 Fast Real-Time PCR System (Applied Biosystems) for the quantification of calpain-1, calpastatin myoglobin, and myosin heavy chain isoforms. Relative gene expression was quantified by the 2^–ΔΔCt^ method.

**Table 2.** Primer sequence used in quantitative reverse transcription—PCR assays

Gene name	Sequence
*MHC I*	F5: AAA-GCT-AGC-CCA-GCT-GAT-TAC
	R5: CTC-TCT-CCT-CTC-CAC-CAT-CTT
*MHC IIA*	F1: TCT-GAA-CTC-TGC-TGA-CCT-ACT-C
	R1: CTG-CAT-TGG-TTA-CCT-GCT-CTA-C
*MHC IIX*	F5: AAA-GCT-AGC-CCA-GCT-GAT-TAC
	R5: CTC-TCT-CCT-CTC-CAC-CAT-CTT
*Myoglobin*	F1: CAG-GCT-CTT-CAC-AGG-TCA-TC
	R1: CCT-CAT-CTC-AGC-CTC-TGT-CTT-C
*S18*	F5: GCG-AGT-CAA-CAC-CAC-CAA-CAT-C
	R5: CCT-CAA-CAC-CAC-ATG-AGC-ATA-TC

### Mitochondrial DNA Content

Total DNA was purified using a DNAeasy mini spin column according to manufacturer’s recommendation (Quigen, Germantown, MD and quantified by optical density at 260 nm (Nanodrop 2000 spectrophotometer, ThermoScientific, USA). Mitochondria (mtDNA) and genomic DNA (gDNA) quantification were accomplished as previously described (López-Andreo et al., 2005). Briefly, 25 ng of total DNA was amplified (TaqMan Fast Advanced Master Mix Applied Biosystems) with organelle-specific DNA primers (500 nm each) and 250 nM MGB probe for 40 cycles of 20 s at 95 °C and 30 s at 60 °C for 40 cycles. Total mtDNA quantity (ng/μL) was inferred from the standard curve and normalized to the gDNA total quantity presented as a ratio of the 2.

### Feed Sampling and Analysis

Individual ingredients in the finishing diet were sampled before the start of the finishing period and analyzed for DM, crude protein (CP), neutral detergent fiber (NDF), acid detergent fiber, Ca, P, net energy of maintenance (NE_m_), and net energy of gain (NE_g_) by Dairy One (Ithaca, NY). The methods described by Goering and Van Soest (1970) were used to analyze the percent DM of feed samples. Crude protein was analyzed using AOAC (1999) method 990.03 by combusting grounds samples with a CN928 Carbon/Nitrogen Determinator (Leco Corporation, St. Joseph, MI). Detergent fiber was analyzed using a Delta Fiber Analyzer (ANKOM Technology, Macedon, NY). The acid detergent fiber was run using ANKON Technology Method 14 and AOAC (1999) method 973.18. Neutral detergent fiber was run using the solutions described by Van Soest et al. (1991) and ANKON Technology Method 15. Mineral were analyzed by processing samples with a MARS6 microwave digester (CEM Corporation, Matthews, NC) and by quantifying mineral concentration using a Thermo iCAP Pro XP Inductively Coupled Plasma Radial Spectrometer (Thermo Fisher Scientific, Waltham, MA). Net energy values were calculated by Dairy One using the OARDC Summative Energy Equation that uses analyzed and book values for CP, NDF, ether extract, ash, lignin, acid detergent insoluble CP, and neutral detergent insoluble CP values.

### Statistical Analysis

Data were analyzed using the Proc Mixed procedure using SAS version 9.3 (SAS Institute Inc., Cary, NC, USA). Because steers were reared and fed in treatments grouped paddocks, and the focus of this work was on postmortem quality attributes, differences are likely not a result of pen or intake. Therefore, carcass was considered the experimental unit (Gootwine, et al., 2020; Wicks et al., 2024) The statistical model included the fixed effect of treatment, with harvest group included as a random variable of harvest date for measures of live performance that included final BW (final BW, overall finishing ADG, and day 30 to harvest ADG) as well as all measures of carcass evaluation, pH analysis, protein abundance, gene expression, and mtDNA. A repeated measures statement was used for pH analysis. Color data was analyzed using fixed effects of treatment and aging time (d); with loin included as a random variable. Means were compared using the Tukey–Kramer Multiple Comparison Test if a significant effect was detected. Data on graphs figures are presented as least square means ± standard error means (SEM), and differences were considered significant at *P* < 0.05 unless otherwise stated.

## Results

### Animal Growth Performance and Carcass Evaluation

Live animal performance during the backgrounding and finishing periods are presented in Table 3. There were no treatment differences (*P* = 0.605) in initial backgrounding BW or backgrounding ADG (*P* = 0.923). Steer BW on day 0 of the finishing period was also not different (*P* = 0.794) between treatments. Grain-fed cattle (LF) had greater (*P* < 0.001) ADG than pasture-managed treatments (CON and SF) during the first 30 d of the finishing period, resulting in LF having greater (*P* = 0.048) BW on day 30 than SF with CON being intermediate and not different from either. During the 30 d directly after SF steers were placed on a grain-finishing diet (days 30 to 60 of finishing period), both SF and LF had greater (*P* = 0.001) ADG than CON; however, LF steers remained heavier (*P* = 0.020) than either SF or CON cattle at day 60. At day 86 of the finishing period, LF steers had greater (*P* = 0.011) BW than CON with SF being intermediate and not different from either. Grain-fed treatments grew faster (*P* < 0.001) than CON from day 30 of the finishing period through harvest. All treatments were different (*P* < 0.001) from each other for overall ADG during the finishing period with LF steers being greater (*P* = 0.015) than SF and SF being greater (*P* = 0.001) than CON. Grain-finished treatments had greater (*P* < 0.001) final BW than pasture-finished CON steers.

**Table 3.** Means for steer BW and ADG during backgrounding and finishing period

	Treatments^1^				
Item	CON	SF	LF	SEM	*P*-value
BW, kg					
Initial Backgrounding	267.2	261.0	257.2	7.0	0.605
Day 0 of finishing period^2^	448.8	438.0	439.6	12.1	0.794
Day 30 of finishing period	474.2^ab^	455.4^b^	497.3^a^	11.4	0.048
Day 60 of finishing period	516.8^b^	513.5^b^	557.8^a^	11.6	0.020
Day 86 of finishing period	541.9^b^	556.2^ab^	595.0^a^	11.9	0.011
Final^2^	535.7^b^	593.2^a^	642.0^a^	14.7	<0.001
ADG, kg/d					
Backgrounding	0.848	0.827	0.851	0.046	0.923
Overall Finishing	0.679^c^	1.220^b^	1.620^a^	0.094	<0.001
Days 0 to 30	0.844^b^	0.580^b^	1.923^a^	0.176	<0.001
Days 30 to 60	1.420^b^	1.935^a^	2.016^a^	0.107	0.001
Days 30 to harvest	0.622^b^	1.405^a^	1.503^a^	0.104	<0.001

^1^Control (CON) cattle were grazed on tall fescue pastures during a 127 ± 6 d finishing period. Short-fed (SF) cattle were moved from pasture on day 30 of finishing period and placed on a grain-based finishing diet for 98 ± 8 d (128 ± 8 d total finishing period). Long-fed (LF) cattle were fed a grain-based finishing diet from days 0 to 125 ± 6 of finishing period and to SF cattle from day 30 of finishing period to harvest.

^2^Body weight recorded on day 0 of finishing period used as final backgrounding BW when calculating backgrounding ADG.

^3^Body weight recorded on day before slaughter before steers transported to Virginia Tech Meat Center.

^a,b,c^Treatment means lacking common letters differ *P* < 0.05.

^x,y,z^Treatment means lacking common letters differ 0.05 ≤ *P* ≤ 0.10.

**Figure 2.**
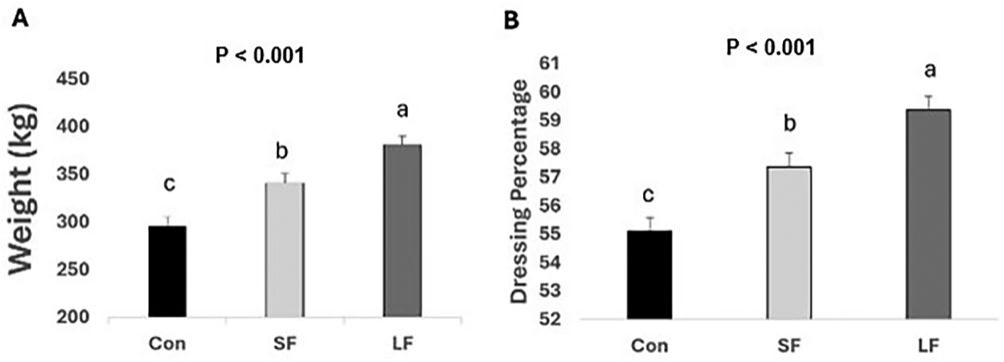
(A) Means for HCW (kg), and (B) dressing percentage (%). Data represent LS means ± SE. Means are considered significantly different at *P* < 0.05. Means lacking common letters differ.

**Figure 3.**
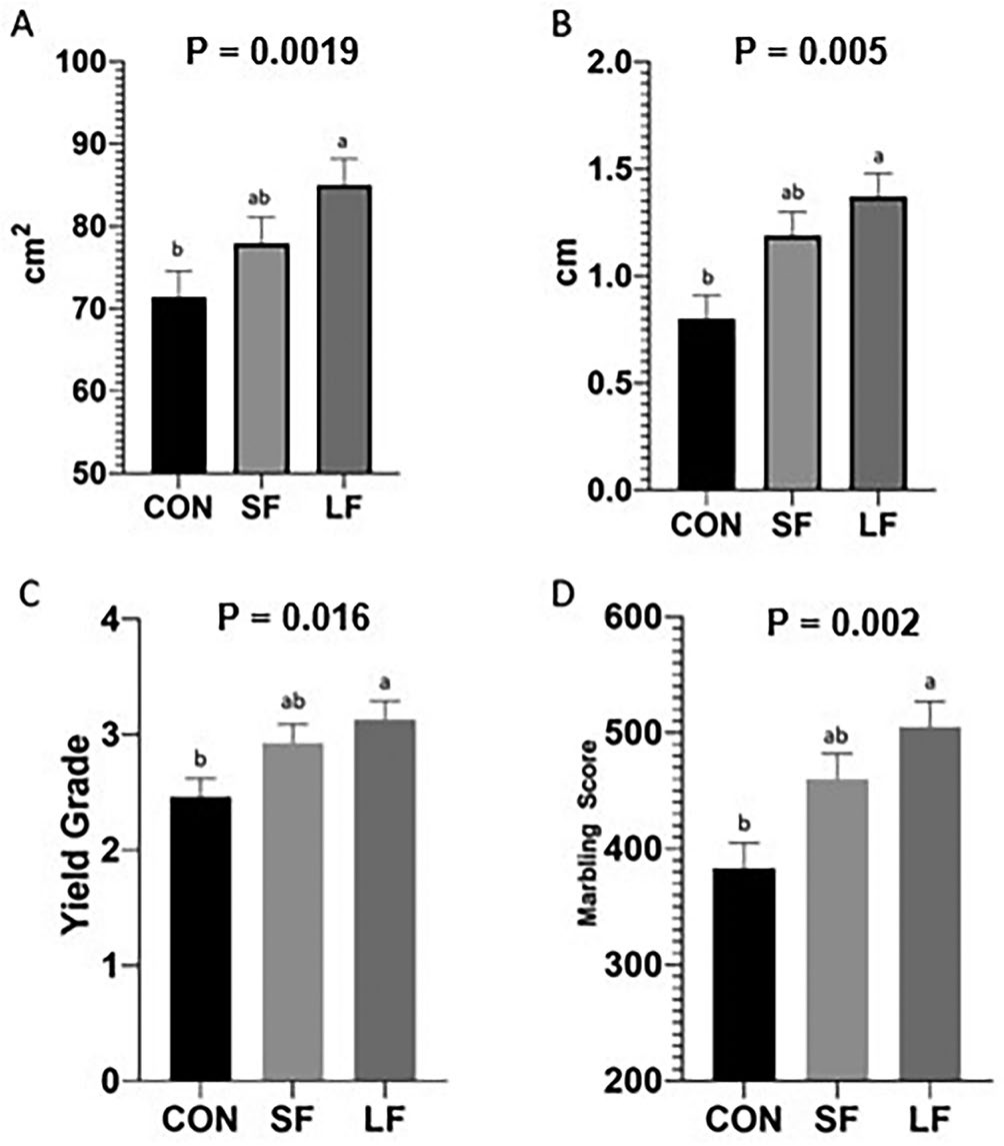
(A) Means for ribeye area (cm^2^), (B) 12th rib fat thickness (cm), (C) yield grade, and (D) marbling score (200 = traces, 300 = slight, 400 = small, 500 = modest, 600 = moderate) between treatments. Data represent LS means ± SE. Means are considered significantly different at *P* < 0.05. Means lacking common letters differ.

Hot carcass weight was increased (*P* < 0.001) with days on feed (Fig. 2A). Dressing percentage was higher in LF compared to CON cattle (*P* < 0.001; Fig. 2B). Additionally, LF carcasses had greater REA (Fig. 3A; *P* = 0.019), FT (Fig. 3B; *P* = 0.005), yield grades (Fig. 3C; *P* = 0.016), and marbling scores (Fig. 3D; *P* = 0.002) compared to those of CON cattle but did not differ from SF. No differences in KPH were noted among treatments (data not shown).

### Color and pH

Lightness (L* value) differed with treatment (*P* = 0.042) and day (*P* < 0.001). Additionally, an interaction between treatment and aging time (d) was noted (Table 4; *P* = 0.0006). Short-fed and LF achieved peak lightness values by day 10 and sustained through day 21. Although lean color from CON carcasses reached similar lightness values to that of LF even after 21 d of aging, lightness was still improved with aging time. In fact, by day 21 there were no longer any differences in L* values between CON and SF. Moreover, CON and SF resulted in the highest a* (redness) values on days 3, 10, 14, and 21 with differences noted on day 3 between CON and LF (Table 4; *P* < 0.0004). While treatments did not vary for yellowness (b*; *P* = 0.595), there was a trend for an interaction between treatment and aging time (Table 4; *P* =* *0.057).

**Table 4.** Means of lightness (L*), redness (a*), and yellowness (b*) values of LL at over 72 h display between treatments

Day	1			3			7			10			14			21				
TRT	CON	SF	LF	CON	SF	LF	CON	SF	LF	CON	SF	LF	CON	SF	LF	CON	SF	LF	SEM	*P*-value
L*	39.03^j^	40.37^fghij^	40.00^hjk^	39.99^ik^	40.86^fghi^	41.53^defg^	39.12^j^	40.49^fghi^	41.29^defgi^	40.22^gik^	41.71^be^	42.60^abc^	41.15^defh^	42.27^acd^	42.54^abc^	41.35^cdefh^	41.88^abcd^	42.74^ab^	0.475	0.0006
a*	22.11^k^	23.31^fghi^	22.63^ijk^	24.52^abcd^	24.37^cde^	23.50^efgh^	22.72^hij^	22.95^ghijk^	22.14^jk^	24.19^bdef^	24.02^cde^	23.74^defg^	24.52^abcd^	24.41^cde^	23.85^defg^	24.80^ac^	25.00^ab^	24.54^abc^	0.327	0.0004
b*	12.54^hi^	13.20^fg^	12.84^gh^	14.12^abcde^	14.12^cde^	13.76^ef^	11.92^j^	12.22^ij^	11.82^j^	13.80^e^	13.81^de^	13.86^de^	14.44^abc^	14.17^cde^	14.07^bde^	14.33^abcd^	14.60^ab^	14.47^ac^	0.199	0.057

Data represent LS means ± SE. Means are considered significantly different at *P <* 0.05. Means lacking common letters within row differ.

**Figure 4.**
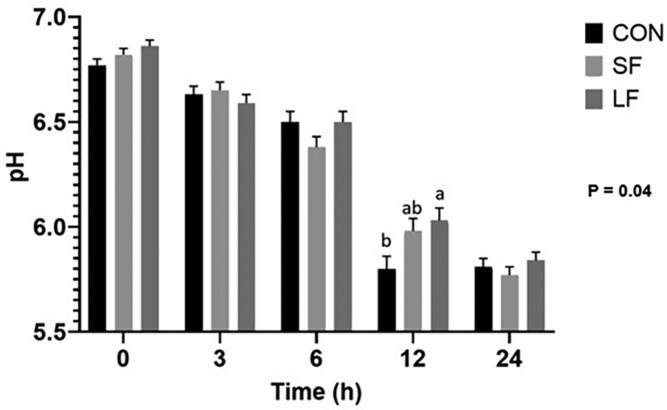
Means of pH values from the LL at across time postmortem. Data represent LS means ± SE. Means are considered significantly different at *P* < 0.05. Means lacking common letters differ.

Despite color variation between treatments and day, ultimate pH (pH_u_) did not differ among treatments. Even so, the rate of pH decline differed with CON being lower at 12 h postmortem (Fig. 4; *P* = 0.04).

### Protein Abundance

**Figure 5.**
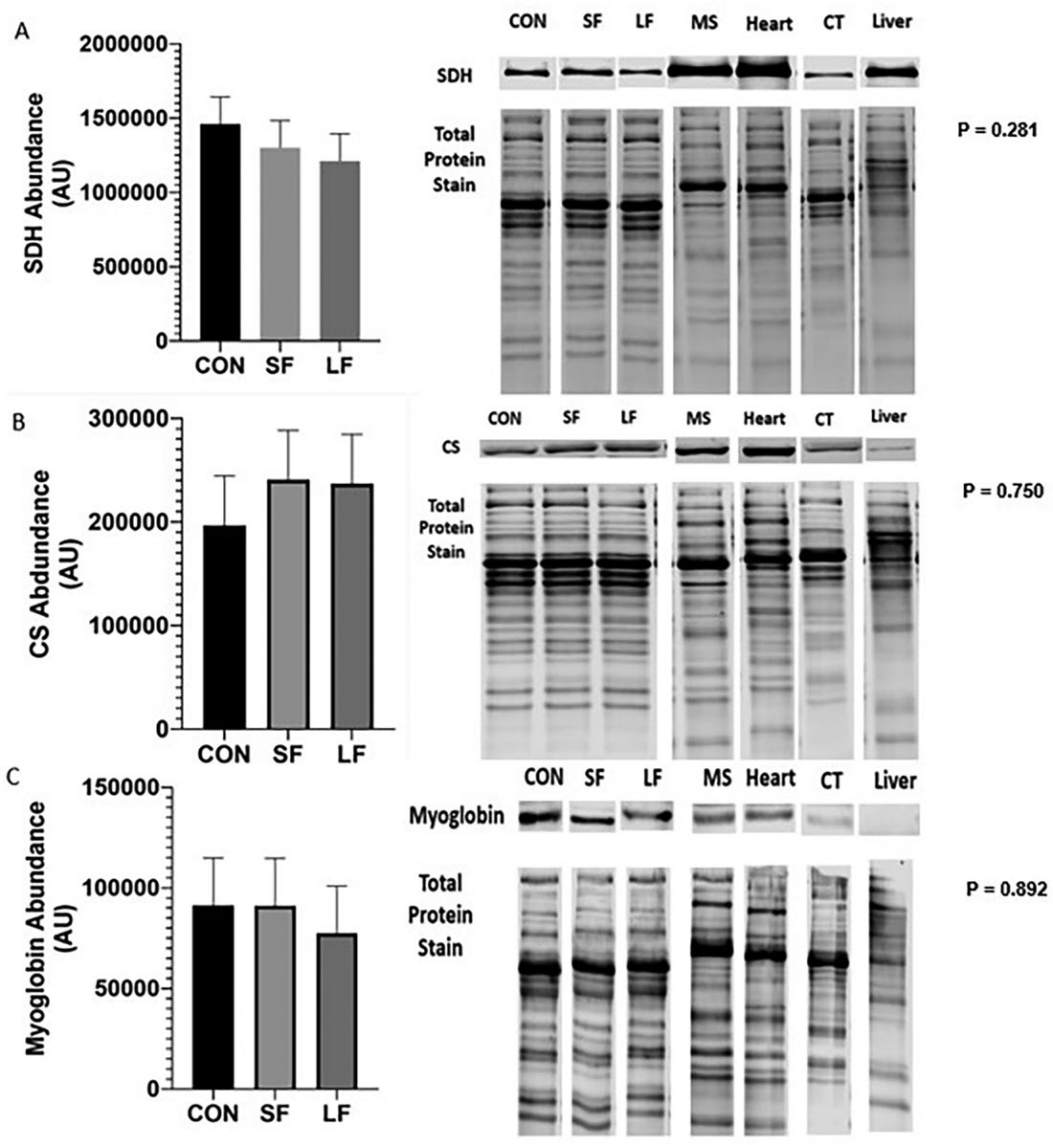
(A) Relative abundance of succinate dehydrogenase (SDH), (B) CS, and (C) myoglobin in *longissimus muscle* (LL) of steers subjected to CON, SF, and LF feeding regimes. Data represent LS means ± SE. Means are considered significantly different at *P* < 0.05.

**Figure 6.**
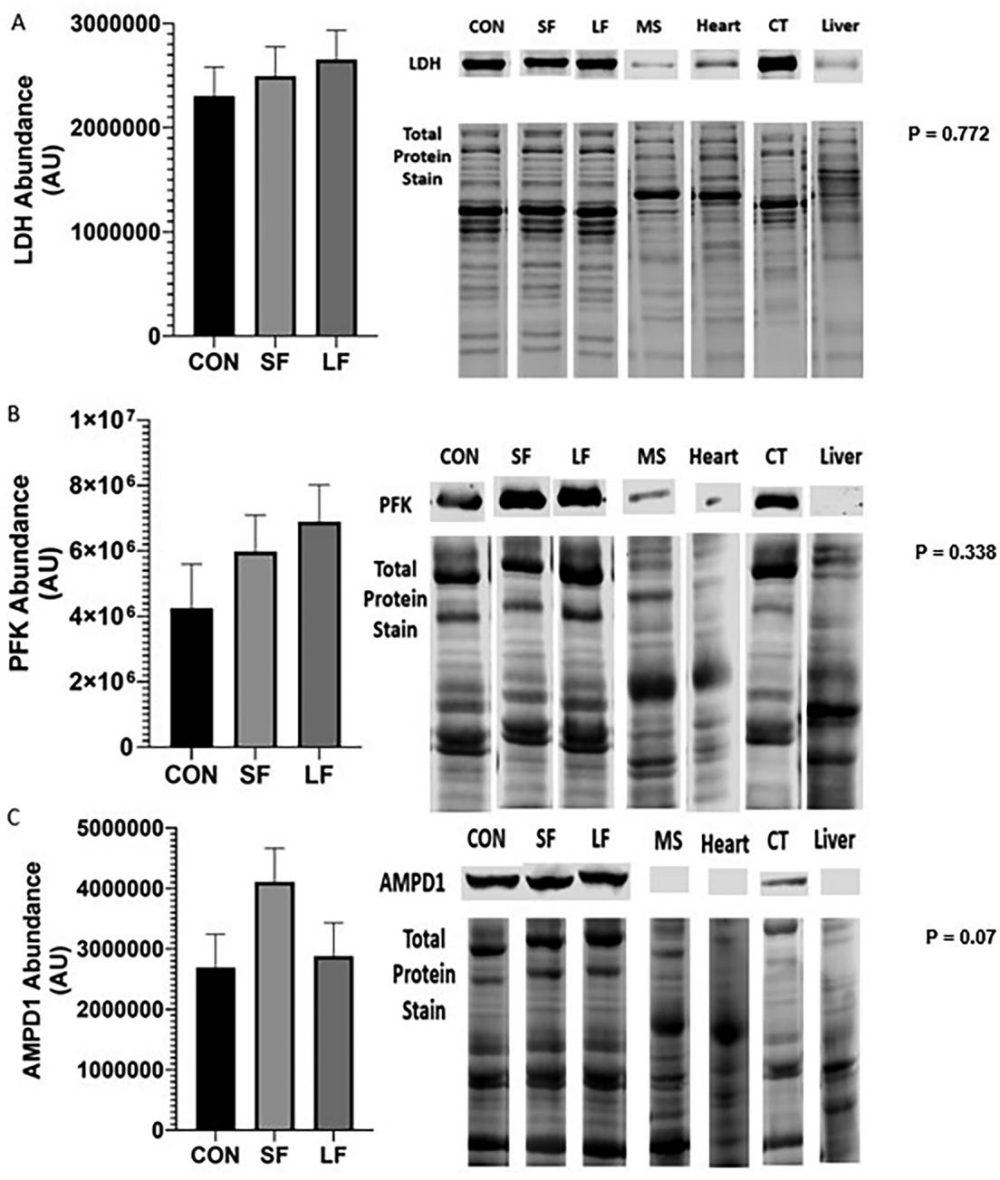
(A) Relative abundance of LDH dehydrogenase (LDH), (B) phosphofructokinase (PFK), and (C) adenosine monophosphate deaminase 1 (AMPD1) in longissimus muscle (LL) of steers subjected to CON, SF, and LF feeding regimes. Data represent LS means ± SE. Means are considered significantly different at *P* < 0.05.

An increase in oxidative muscle proteins such as SDH, CS, and myoglobin has been shown to increase in cattle fed lower energy diets, resulting in increased ultimate pH (pH_u_) and darker lean (Apaoblaza et al., 2020; Gómez et al., 2022). However, we were unable to detect any differences in SDH (Fig. 5A; *P* = 0.281), CS (Fig. 5B; *P* = 0.750), or myoglobin (Fig. 5C; *P* = 0.892) despite differences in feeding regimes. Additionally, we were also unable to distinguish any differences in LDH (Fig. 6A; *P* = 0.772), PFK (Fig. 6B; *P* = 0.338), or AMPD1 (Fig. 6C; *P* = 0.07) all indicators of more glycolytic metabolism.

### Gene Expression and mtDNA

**Figure 7.**
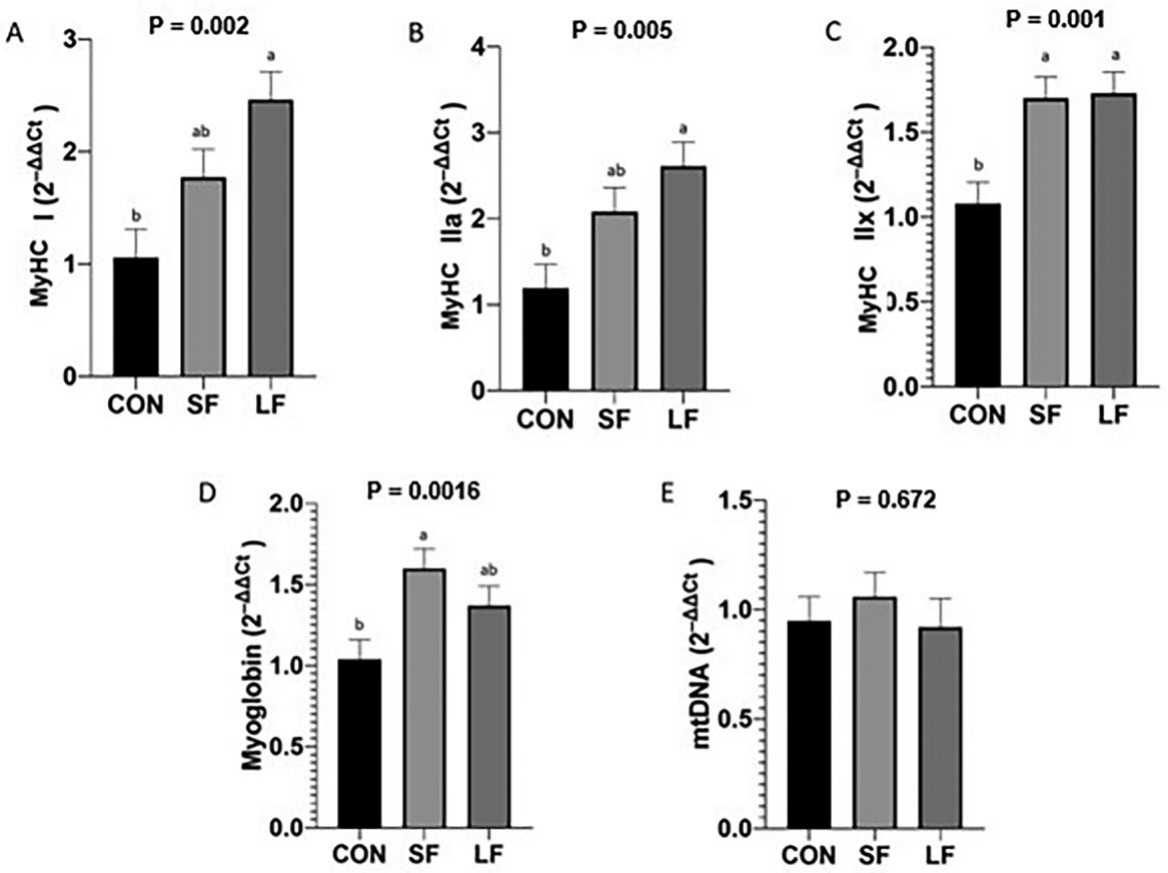
Means of gene expression of (A) myosin heavy chain type I (MyHC-I), (B) myosin heavy chain type IIA (MyHC-IIA), and (C) myosin heavy chain type IIX (MyHC-IIX). (D) Mitochondrial (mt) DNA copy number relative to genomic (g) DNA copy number and (E) myoglobin gene expression between treatments and presented as fold differences. Data represent LS means ± SE. Means are considered significantly different at *P* < 0.05.

Although we were unable to detect differences in protein abundance for myoglobin, there was a significant difference in gene expression of myoglobin in SF cattle compared to CON (Fig. 7D; *P* = 0.016). Expression of MyHC differed between treatments. Long-fed cattle had increased MyHC-I (Fig. 7A; *P* = 0.002), MyHC-IIA (Fig. 7B; *P* = 0.005), MyHC-IIX (Fig. 7C*; P* = 0.001) compared to CON, however, SF cattle did not differ from that of LF cattle. Still, no differences were noted in mtDNA between any treatment (Fig. 7E*; P* = 0.672).

## Discussion

Beef carcasses deemed “dark-cutting” receive one of the largest discounts of any quality designation (USDA-AMS, 2024). While not all grass-fed beef is considered dark-cutting on the grading rail, this scenario conveys the importance of beef color on product marketability. Consumers consider in fresh beef color an indicator of freshness and quality (Dikeman, 1990; Troy and Kerry, 2010), and as such, is often used by consumers as a benchmark when making purchasing decisions, preferring beef that is bright-cherry red. While many factors influence lean color development, nutrient energy source and degree of finish play an intricate role fresh beef color (Vestergaard et al., 2002; Shibata et al., 2009; Apaoblaza et al., 2020; Gómez et al., 2022). Feeding cattle high-forage diets results in greater concentrations of ruminal acetate, while cattle fed high-concentrate diets have relatively higher concentrations of ruminal propionate (Zhang et al. 2017). Utilization of acetate and propionate as energy sources differ, giving rise to 2 different means of energy production. Acetate is a 2-carbon short-chain fatty acid that can be readily converted to acetyl-CoA and is metabolized through the mitochondria to produce energy through the citric acid cycle (Yoshii et al., 2015). On the other hand, propionate primarily functions in the liver as a major substrate for gluconeogenesis, which is predominately used to stabilize blood glucose levels, for the brain, erythrocytes, and kidneys (Aschenbach et al., 2010). Therefore, as substrate availability shifts, so does muscle fiber type, metabolism, and the prospect of the lean color.

Lean color blooming (initial) and stability (sustained) are driven by 2 competing mechanisms. Blooming occurs when oxygen diffuses into the meat. As oxygen penetrates the muscle, it binds to myoglobin converting it to oxymyoglobin, shifting the visual appearance from purple to red. However, because mitochondria are still viable postmortem, and consuming oxygen (OCR; Lanari & Cassens, 1991), there is competition for oxygen in the tissue between the mitochondria and myoglobin. Because the affinity for oxygen is greater for the heme group in cytochrome C, functioning mitochondria limits potential for bloom intensity. On the other hand, color stability is dependent on metmyoglobin reduction activity and relies heavily on mitochondria’s ability to process both enzymatic and nonenzymatic NADH in order to maintain ferrous myoglobin forms. Since grass-fed beef tends to have increased oxidative muscle properties, it is likely their ability to bloom is impeded due to increased mitochondrial function (Apaoblaza et al., 2020). Knecht et al. (2021) determined the affinity of bloom color is dependent on muscle type, showing greatest increase in color (L*) of more glycolytic muscles. Even so, the process of aging allows for the degradation of cellular mechanisms that play an intricate role in governing myoglobin redox chemistry and may be advantageous in increasing bloom and lightness of grass-fed beef. Therefore, we chose to investigate the effects of dry aging on color development of cattle subjected to differing feeding regimes.

Data presented herein provide encouraging and novel insight into fresh beef color in grass-fed cattle. First, our data show color (L*, a*, b*) is improved with dry aging, regardless of treatment (Table 4). Though not the objective of the Gómez et al. (2022) study, they also reported improvements in lightness with aging in both grass and grain-fed beef. However, our data are unique in that they show a threshold in which lean color can be improved. Our data show that fed cattle (LF, SF) achieved ultimate lightness and redness by 10 d, which was maintained through 21 d. Conversely, CON required 14 d to reach its ultimate lightness. Redness (a*) appeared to vary with treatment and day. Still, a* was greatest on day 21, as was b* value. It is well-established that mitochondrial oxygen consumption decreases with aging thus minimizing competition of oxygen between the mitochondria and myoglobin, consequently improving initial (bloom) color. Our data supports this thesis. However, to better understand these results and the impact of diet on muscle and its subsequent implication to lean color, we evaluated both carcass and metabolic biomarkers. Specifically, our data show differences in BWs and carcass parameters between CON and LF cattle (Figs. 2A, B and 3A, D), and indicate differences in growth and degree of finish, which may be lead to changes in metabolism favoring muscle from CON cattle compared to that of the LF cattle (Lefaucheur, 2010; Koch et al., 2019; Apaoblaza et al, 2020; Antonelo et al., 2022; Gómez et al., 2022). This is further supported by our data which show differences between CON and LF in gene expression of MyHC-IIA, and -IIX (Fig. 7B and C) suggesting muscle from LF cattle have a greater glycolytic phenotype. Even so, we found no difference in mtDNA between treatments (Fig. 7E), though fast-twitch glycolytic fibers tend to have fewer mitochondria than slow oxidative fibers (Leary et al., 2003). This lack of relationship is counter to our proposition that grain-fed cattle have more glycolytic muscle but Apaoblaza et al. (2020) also failed to detect differences in mtDNA abundance. However, unlike our data which showed no evidence of differences in enzymatic properties between grass and grain-fed tissue (Figs. 5A to C and 6A to C), Apaoblaza et al. (2020) detected significant differences in the oxidative proteins SDH and myoglobin between grass and grain-fed muscle, raising the possibility that differences in mitochondrial function in muscle change with diet (Bai et al., 2020), further complicating mechanisms responsible for disparities in lean color development. Even so, lean color is not predicated solely on muscle type. In fact, pH greatly influences lean color, especially in regard to lightness (reviewed by Ramanathan et al., 2020). Typically, pH gradually declines from a pH of approximately 7.0 to an ultimate pH of 5.5 to 5.6 giving beef the classic bright-cherry red color that consumers prefer (reviewed by Boles and Pegg, 2010). Our data show pH_u_ to be slightly higher than 5.6 across all treatments (Fig. 4), yet differences in color among treatments exist with that of CON cattle resulting in the darkest lean. Despite a lack of differences in pH_u_, our results showed a significant decline between 6 and 12 h after harvest for CON cattle, leading to significantly lower pH at 12 h for CON compared to the fed groups. Although somewhat ambiguous, a difference in the rate of pH decline implies a difference in metabolism between CON and fed groups. Moreover, the pH of CON muscle postmortem remained fairly constant between 12 and 24 h. This indicates a difference in muscle metabolism may contribute to the prolonged aging time required for CON treatment to achieve a similar lightness of lean to that of its fed counterparts.

## Conclusion

Overall, these data reaffirm that grass-fed beef is darker than of grain-fed beef, regardless of the extent to which cattle are on feed. Still, our data raise questions about the underlying mechanism that controls lean color development because we were unable to replicate the results of Apaoblaza et al. (2020) or Antonelo et al. (2022) which determined darker lean arises due to a more oxidative phenotype. Still, dry aging improved lean color up to 21 d regardless of treatment. However, our data showed thresholds in which beef reaches peak lightness. Fed cattle achieve an ultimate lightness (L*) following a 10-d aging period, however, grass-fed beef appears to require additional aging time, closer to 14 d suggesting these cattle had altered mitochondrial function, which changed with aging, thus allowing for greater oxygenation of myoglobin. While further work is needed in terms of long-term display and tenderization through the practice of dry aging, our data argue extended aging past 14 d may not be necessary in order to achieve optimal quality and could result in increased processing capacity with faster turnover time. Moreover, our data also argue grass-fed beef, though initially darker than that of grain-fed, can achieve a similar lean color when dry aged.

## References

[CIT0001] American Meat Science Association (AMSA), National Cattlemen’s Beef Association (US), and National Pork Producers Council (US). 2001. Meat evaluation handbook. Champaign (IL): Amer Meat Science Assn.

[CIT0002] Antonelo, D. S., J. F. Gómez, S. L. Silva, M. Beline, X. Zhang, Y. Wang, B. Pavan, L. A. Koulicoff, A. F. Rosa, R. S. Goulart, et al 2022. Proteome basis for the biological variations in color and tenderness of longissimus thoracis muscle from beef cattle differing in growth rate and feeding regime. Food Res. Int. 153:110947. doi: https://doi.org/10.1016/j.foodres.2022.110947 35227471

[CIT0003] AOAC. 1999. Official methods of analysis. 16th ed. Assoc. Off. Anal. Chem., Washington, D.C.

[CIT0004] Aschenbach, J. R., N. B. Kristensen, S. S. Donkin, H. M. Hammon, and G. B. Penner. 2010. Gluconeogenesis in dairy cows: the secret of making sweet milk from sour dough. IUBMB Life 62:869–877. doi: https://doi.org/10.1002/iub.400 21171012

[CIT0005] Apaoblaza, A., S. D. Gerrard, S. K. Matarneh, J. C. Wicks, L. Kirkpatrick, E. M. England, T. L. Scheffler, S. K. Duckett, H. Shi, S. L. Silva, et al 2020. Muscle from grass-and grain-fed cattle differs energetically. Meat Sci. 161:107996. doi: https://doi.org/10.1016/j.meatsci.2019.107996 31734468

[CIT0006] Bai, Y., J. A. Carrillo, Y. Li, Y. He, and J. Song. 2020. Diet induced the change of mtDNA copy number and metabolism in Angus cattle. J. Anim. Sci. Biotechnol. 11:1–13. doi: https://doi.org/10.1186/s40104-020-00482-x 32699629 PMC7372754

[CIT0007] Bauman P. 2021. Grass-fed beef: market share of grass-fed beef. [accessed May 28, 2023]. https://extension.sdstate.edu/grass-fed-beef-market-share-grass-fed-beef

[CIT0008] Bendall, J. R. 1973. Postmortem changes in muscle. The structure and function of muscle. Vol. 2. Academic Press, New York. p. 243243–309309.

[CIT0009] Brewer, P., and C. R. Calkins. 2003. Quality traits of grain-and grass-fed beef: a review. Nebraska Beef Cattle Rep. 221:74–77.

[CIT0010] Boles, J. A., and R. Pegg. 2010. Meat color. Saskatchewan (SK): Montana State University and Saskatchewan Food Product Innovation, Program University of Saskatchewan. Retrieved from: https://safespectrum.com/wp-content/uploads/2023/09/meatcolor.pdf

[CIT0011] Chail, A., J. F. Legako, L. R. Pitcher, T. C. Griggs, R. E. Ward, S. Martini, and J. W. MacAdam. 2016. Legume finishing provides beef with positive human dietary fatty acid ratios and consumer preference comparable with grain-finished beef. J. Anim. Sci. 94:2184–2197. doi: https://doi.org/10.2527/jas.2015-0241 27285714

[CIT0012] Chase, S. 2020. Report: Grass-fed beef market could grow by $14B by 2024. [accessed April 4, 2021]. https://www.agri-pulse.com/articles/14645-report-grass-fed-beef-market-could-grow-by-14b-by-2024

[CIT0013] Crouse, J. D., H. R. Cross, and S. C. Seideman. 1984. Effects of a grass or grain diet on the quality of three beef muscles. J. Anim. Sci. 58:619–625. doi: https://doi.org/10.2527/jas1984.583619x

[CIT0014] Dikeman, M. E. 1990. Genetic effects on the quality of meat from cattle. In Proceedings of the 4th World Congress on Genetics applied to Livestock Production. Edinburgh 23-27 July 1990. XV. Beef cattle, sheep and pig genetics and breeding, fibre, fur and meat quality. Edinburgh (UK): Cabi Digital Library; p. 521–530. Retrieved From: cabidigitallibrary.org/doi/full/10.5555/19900183325

[CIT0015] Davis, G. W., A. B. Cole Jr, W. R. Backus, and S. L. Melton. 1981. Effect of electrical stimulation on carcass quality and meat palatability of beef from forage-and grain-finished steers. J. Anim. Sci. 53:651–657. doi: https://doi.org/10.2527/jas1981.533651x

[CIT0017] FASS. 2020. Guide for the care and use of agricultural animals in research and teaching. 4th ed. Fed. Anim. Sci. Soc., Champaign, IL.

[CIT0018] Goering, H. K. & Van Soest, P. J. 1970. Forage fiber analyses: apparatus, reagents, procedures, and some applications. USDA-ARS Agricultural Handbook 379, Washington, D.C.

[CIT0019] Gómez, J. F. M., D. S. Antonelo, M. Beline, B. Pavan, D. B. Bambil, P. Fantinato-Neto, A. Saran-Netto, P. R. Leme, R. S. Goulart, D. E. Gerrard, et al 2022. Feeding strategies impact animal growth and beef color and tenderness. Meat Sci. 183:108599. doi: https://doi.org/10.1016/j.meatsci.2021.108599 34365253

[CIT0020] Gootwine, E., A. Rosov, T. Alon, C. Stenhouse, K. M. Halloran, G. Wu, and F. W. Bazer. 2020. Effect of supplementation of unprotected or protected arginine to prolific ewes on maternal amino acids profile, lamb survival at birth, and pre-and post-weaning lamb growth. J. Anim. Sci. 98:skaa284. doi: https://doi.org/10.1093/jas/skaa284 32860700 PMC7694597

[CIT0021] Holman, B. W., A. E. D. A. Bekhit, Y. Mao, Y. Zhang, and D. L. Hopkins. 2022. The effect of wet ageing duration (up to 14 weeks) on the quality and shelf-life of grass and grain-fed beef. Meat Sci. 193:108928. doi: https://doi.org/10.1016/j.meatsci.2022.108928 35930968

[CIT0045] Koch, B. M., E. Pavan, N. M. Long, J. G. Andrae, and S. K. Duckett. 2019. Postweaning exposure to high concentrates versus forages alters marbling deposition and lipid metabolism in steers. Meat and Muscle Biology 3:244. doi: https://doi.org/10.22175/mmb2018.12.0040

[CIT0022] Knecht, D., K. Duziński, and A. Jankowska-Mąkosa. 2021. Bloom time effect depends on muscle type and may determine the results of pH and color instrumental evaluation. Animals. 11:1282. doi: https://doi.org/10.3390/ani11051282 33947084 PMC8146694

[CIT0023] Lanari, M. C., and R. G. Cassens. 1991. Mitochondrial activity and beef muscle color stability. J. Food Sci. 56:1476–1479. doi: https://doi.org/10.1111/j.1365-2621.1991.tb08619.x

[CIT0024] Leary, S. C., C. N. Lyons, A. G. Rosenberger, J. S. Ballantyne, J. Stillman, and C. D. Moyes. 2003. Fiber-type differences in muscle mitochondrial profiles. Am. J. Physiol. Regul. Integr. Comp. Physiol. 285:R817–R826. doi: https://doi.org/10.1152/ajpregu.00058.2003 12947029

[CIT0025] Lefaucheur, L. 2010. A second look into fibre typing–relation to meat quality. Meat Sci. 84:257–270. doi: https://doi.org/10.1016/j.meatsci.2009.05.004 20374784

[CIT0026] López-Andreo, M., L. Lugo, A. Garrido-Pertierra, M. I. Prieto, and A. Puyet. 2005. Identification and quantitation of species in complex DNA mixtures by real-time polymerase chain reaction. Anal. Biochem. 339:73–82. doi: https://doi.org/10.1016/j.ab.2004.11.045 15766713

[CIT0029] Martin, J. M., and R. W. Rogers. 2004. Forage-produced beef: challenges and potential. Prof. Anim. Sci. 20:205–210. doi: https://doi.org/10.15232/s1080-7446(15)31302-4

[CIT0030] NASEM. 2016. Nutrient requirements of beef cattle. 8th Revised ed. Washington (DC): The National Academies Press. doi: https://doi.org/10.17226/19014

[CIT0031] Ramanathan, R., S. P. Suman, and C. Faustman. 2020. Biomolecular interactions governing fresh meat color in post-mortem skeletal muscle: a review. J. Agric. Food Chem. 68:12779–12787. doi: https://doi.org/10.1021/acs.jafc.9b08098 32045229

[CIT0033] Shibata, M., K. Matsumoto, M. Oe, M. Ohnishi-Kameyama, K. Ojima, I. Nakajima, K. Muroya, and K. Chikuni. 2009. Differential expression of the skeletal muscle proteome in grazed cattle. J. Anim. Sci. 87:2700–2708. doi: https://doi.org/10.2527/jas.2008-1486 19420231

[CIT0034] Suman, S. P., M. C. Hunt, M. N. Nair, and G. Rentfrow. 2014. Improving beef color stability: practical strategies and underlying mechanisms. Meat Sci. 98:490–504. doi: https://doi.org/10.1016/j.meatsci.2014.06.032 25041654

[CIT0035] Troy, D. J., and J. P. Kerry. 2010. Consumer perception and the role of science in the meat industry. Meat Sci. 86:214–226. doi: https://doi.org/10.1016/j.meatsci.2010.05.009 20579814

[CIT0036] USDA-AMS. 2024. National weekly direct slaughter-cattle – premiums and discounts. [accessed June 5, 2023]. https://usda.library.cornell.edu/concern/publications/m326m1803?locale=en

[CIT0037] Van Soest, P. J., J. B. Robertson, and B. A. Lewis. 1991. Methods for dietary fiber, neutral detergent fiber, and nonstarch polysaccharides in relation to animal nutrition. J. Dairy Sci. 74:3583–3597. doi: https://doi.org/10.3168/jds.S0022-0302(91)78551-2 1660498

[CIT0038] Veiseth, E., and M. Koohmaraie. 2005. Beef tenderness: significance of the calpain proteolytic system. In Indicators of milk and beef quality. Wageningen (Netherlands): Wageningen Academic. p. 111–126. doi: https://doi.org/10.3920/9789086865376_009

[CIT0044] Vestergaard, M., N. Oksbjerg, and P. Henckel. 2002. Influence of feeding intensity, grazing and finishing feeding on muscle fibre characteristics and meat colour of semitendinosus, longissimus dorsi and supraspinatus muscles of young bulls. Meat Sci. 54:177–185. doi: https://doi.org/10.1016/S0309-1740(99)00097-2 22060614

[CIT0039] Warren, C. M., P. R. Krzesinski, and M. L. Greaser. 2003. Vertical agarose gel electrophoresis and electroblotting of high‐molecular‐weight proteins. Electrophoresis. 24:1695–1702. doi: https://doi.org/10.1002/elps.200305392 12783444

[CIT0042] Wicks, J., M. Beline, J. F. M. Gomez, S. Luzardo, S. L. Silva, and D. Gerrard. 2019. Muscle energy metabolism, growth, and meat quality in beef cattle. Agriculture 9:195–195. doi: https://doi.org/10.3390/agriculture9090195

[CIT0043] Wicks, J. C., A. L. Wivell, M. Beline, M. D. Zumbaugh, J. S. Bodmer, C.-N. Yen, C. Johnson-Schuster, T. B. Wilson, S. P. Greiner, S. E. Johnson, et al 2024. Determining muscle plasticity and meat quality development of low-input extended fed market-ready steers. Transl. Anim. Sci. 8:txae064. doi: https://doi.org/10.1093/tas/txae064 38770036 PMC11103109

[CIT0040] Yoshii, Y., T. Furukawa, T. Saga, and Y. Fujibayashi. 2015. Acetate/acetyl-CoA metabolism associated with cancer fatty acid synthesis: overview and application. Cancer Lett. 356:211–216. doi: https://doi.org/10.1016/j.canlet.2014.02.019 24569091

[CIT0041] Zhang, J., H. Shi, Y. Wang, S. Li, Z. Cao, S. Ji, Y. He, and H. Zhang. 2017. Effect of dietary forage to concentrate ratios on dynamic profile changes and interactions of ruminal microbiota and metabolites in Holstein heifers. Front. Microbiol. 8:2206. doi: https://doi.org/10.3389/fmicb.2017.02206 29170660 PMC5684179

